# Genetic structure and diversity of the endangered growling grass frog in a rapidly urbanizing region

**DOI:** 10.1098/rsos.140255

**Published:** 2015-08-26

**Authors:** Claire C. Keely, Joshua M. Hale, Geoffrey W. Heard, Kirsten M. Parris, Joanna Sumner, Andrew J. Hamer, Jane Melville

**Affiliations:** 1 School of BioSciences, University of Melbourne, Parkville 3010, Australia; 2 Australian Research Centre for Urban Ecology, Royal Botanic Gardens Melbourne c/o School of BioSciences, University of Melbourne, Parkville 3010, Australia; 3 Sciences Department, Museum Victoria, Carlton 3053, Australia

**Keywords:** *Litoria raniformis*, urbanization, genetic diversity, haplotype

## Abstract

Two pervasive and fundamental impacts of urbanization are the loss and fragmentation of natural habitats. From a genetic perspective, these impacts manifest as reduced genetic diversity and ultimately reduced genetic viability. The growling grass frog (*Litoria raniformis*) is listed as vulnerable to extinction in Australia, and endangered in the state of Victoria. Remaining populations of this species in and around the city of Melbourne are threatened by habitat loss, degradation and fragmentation due to urban expansion. We used mitochondrial DNA (mtDNA) and microsatellites to study the genetic structure and diversity of *L. raniformis* across Melbourne's urban fringe, and also screened four nuclear gene regions (POMC, RAG-1, Rhod and CRYBA1). The mtDNA and nuclear DNA sequences revealed low levels of genetic diversity throughout remnant populations of *L. raniformis*. However, one of the four regions studied, Cardinia, exhibited relatively high genetic diversity and several unique haplotypes, suggesting this region should be recognized as a separate Management Unit. We discuss the implications of these results for the conservation of *L. raniformis* in urbanizing landscapes, particularly the potential risks and benefits of translocation, which remains a contentious management approach for this species.

## 1. Introduction

Urbanization represents a growing threat to the conservation of biodiversity. The impacts of urbanization on biodiversity are numerous [1], but a pervasive and fundamental effect is the loss and fragmentation of habitat [2,3]. From a genetic perspective, these impacts manifest as reduced genetic diversity and ultimately reduced genetic viability, as a result of declines in local population size and changes in the rate of dispersal between habitat patches. Populations in small habitat fragments also suffer from edge effects and increased exposure to predators [4], as well as fine-scale changes in microclimate and other attributes that influence habitat quality [5,6]. The resulting small, fragmented populations are susceptible to genetic drift and inbreeding depression [7], both of which can lead to the erosion of genetic diversity and genetic viability [8]. These impacts are a pervasive problem in urban areas and as such, conservation genetics has become an important tool for the management of threatened species in urban landscapes.

Assessing the genetic origins of populations and effectively managing genetic diversity are central to their persistence in both the short and long term [9,10]. Genetic analyses of population structure, dispersal and gene flow can improve our understanding of how species respond to landscape change [11], and hence provide insights into landscape modifications that may reduce isolation and the extinction risk of fragmented populations [12]. Genetic information is also vital for active manipulation of populations fragmented by urban development, such as population augmentation and translocation [13]. In many cases, initiatives such as these seek to restore the fitness of populations exhibiting symptoms of inbreeding depression, by introducing individuals (and hence, genes) from related populations [14]. However, without prior knowledge of the genetic structure of a species, there is a risk of altering the genetic structure of populations [15], potentially causing problems such as outbreeding depression or hybridization of divergent evolutionary lineages [16]. Thus, detailed knowledge of the population genetic structure of a species is essential prior to the initiation of programmes aimed at genetic rescue, such as population augmentation and translocation.

The proportion of the world's human population living in urban areas grew rapidly during the twentieth century, from an estimated 220 million to 2.8 billion [17]. Australia is expected to become the world's fastest growing industrialized nation over the next four decades [18], and Melbourne—the capital city of the state of Victoria—is the fastest growing major city in Australia [19]. The Melbourne Metropolitan Area (MMA) currently covers approximately 7500 km^2^ and has a population of approximately 4.25 million people [20]. Recent expansions of the urban growth boundary will increase the MMA by an additional 400 km^2^ over the next 20 years [21]. Urban growth is expected to negatively impact the genetic diversity and viability of several threatened species in this region [21].

**Figure 1.**
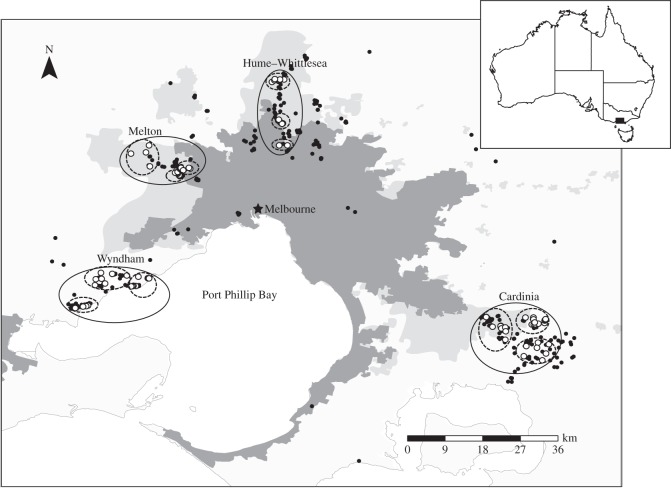
Geographical distribution and sample sites of *L. raniformis* around Melbourne. Dark grey, current MMA; light grey, proposed urban growth area. Closed circles indicate detection records between 2000 and the present. Open circles indicate the location of sampling sites included in this study. The four study regions around Melbourne are indicated and clusters of sites within these regions circled (dashed lines).

In this study, we investigated the conservation genetics of the endangered growling grass frog (*Litoria raniformis*) in the MMA. This species has declined markedly over the last three decades, yet significant remnant populations persist in the low-altitude, urban-fringe environments to the southwest (Wyndham), northwest (Melton), north (Hume–Whittlesea) and southeast (Cardinia) of Melbourne (figure 1). We used mitochondrial DNA (mtDNA: cytochrome oxidase I (COI) and ND4), four nuclear gene regions (POMC, RAG-1, Rhod and CRYBA1) and microsatellite genotyping to investigate the genetic structure and diversity of *L. raniformis* across Melbourne. We predict that: (i) genetic diversity of *L. raniformis* across Melbourne's urban fringe is low due to past declines and more recent habitat loss and fragmentation from urbanization, (ii) a genetic bottleneck may have occurred as a result of these processes, and (iii) genetic structuring is present between remnant populations in Cardinia and the other three regions, due both to the fundamental geographical isolation of this region and to the more recent loss of connecting populations (figure 1).

## 2. Material and methods

### 2.1 Study species

*Litoria raniformis* is a semi-aquatic species that inhabits still or slow-flowing sections of streams, as well as lentic wetlands [22]. This species was abundant across much of southeastern Australia [23]; however, it has declined significantly since the late 1970s and the frog is now listed as endangered [24,25]. Although numerous threatening processes drove this decline, the arrival of the chytrid fungus (*Batrachochytrium dendrobatidis*) in southeastern Australia and resulting epidemics of chytridiomycosis were almost certainly a major contributor [26]. Populations at higher altitude were particularly affected [27], but low-altitude populations survived in areas less suitable for chytrid [28], allowing functioning metapopulations to be maintained, particularly in regions with a high density and connectivity of wetland habitats [29–31].

Despite persisting through the declines last century, remnant populations of *L. raniformis* around Melbourne are now threatened by the rapid pace of urban expansion. Urbanization has led to wetland loss, degradation and fragmentation in each of the four regions in which significant remnant populations of *L. raniformis* persist (Wyndham, Melton, Hume–Whittlesea and Cardinia; figure 1), and this has caused the loss and fragmentation of some populations in recent years [32]. This process will continue over the next three decades, with these regions being designated urban growth areas in which the proposed urban boundaries encompass numerous remaining populations of *L. raniformis* (figure 1) [33].

### 2.2 Study area and field sampling

Tissue samples (*N*=377) were collected from remnant populations of *L. raniformis* in the Wyndham, Melton, Hume–Whittlesea and Cardinia regions (figure 1). One hundred and seven samples were collected from Hume–Whittlesea as part of a previous study [30] (see below). The remaining samples (*N*=270) were collected during the current study between December 2010 and March 2011 across the other three regions. We divided each region into three geographical clusters of sites for the purposes of sampling (12 clusters altogether, figure 1). We chose these clusters to maximize the geographical area sampled within each region, and to encompass networks of sites likely to support metapopulations of *L. raniformis* [30,34]. Sampling sites (*N*=73) included slow-flowing pools along streams, as well as farm dams, swamps and water-treatment ponds. Between 1 and 10 individuals were sampled per site depending on population size and capture success.

Genetic samples were obtained from each frog by clipping a triangular section of toe webbing (approx. 2 mm at the base) between the second and third toes closest to the body on the left hind limb. Toe web samples were stored in 95% ethanol and kept at −18°C for approximately two weeks, then −80°C for up to 3.5 months. Further details on these samples may be found in electronic supplementary material, S1. Latex gloves were worn when taking samples and were changed between individuals. Tissue sampling equipment was also sterilized in 70% ethanol between frogs to prevent the spread of pathogens such as the chytrid fungus.

Samples from Hume–Whittlesea were collected between October 2004 and February 2006 as part of a study on the metapopulation dynamics of *L. raniformis* in this region [30] (figure 1). In that study, frogs were captured by hand during spotlight surveys and tissue samples obtained by clipping the toe-pad on the left middle digit of the front left limb following standard procedures (see [35] for additional details).

### 2.3 DNA extraction and sequencing

We sequenced the mitochondrial gene COI for all 377 samples and a subset of samples (*N*=112) were sequenced for NADH dehydrogenase subunit 4 (ND4) encompassing all COI haplotypes and geographical regions. Each of these gene regions have been shown to be variable in previous studies of *L. raniformis* [36,37]. We also sequenced four nuclear gene regions (POMC, RAG-1, Rhod, CRYBA1 for a subset of 19 samples each, encompassing the extent of mtDNA haplotypic diversity) that are known to be variable at an intraspecific level in frogs [38–41].

Samples collected in the Hume–Whittlesea region were extracted and sequenced for COI as part of a previous, as yet unpublished, study [37]. We sequenced all other gene regions for these samples as part of this study. Samples collected from all other geographical areas were extracted and sequenced for all gene regions during this study. In all cases, genomic DNA was extracted using a DNeasy Blood & Tissue Kit (QIAGEN) using the manufacturer's animal tissue protocol.

We performed PCR amplification of the samples from Wyndham, Melton and Cardinia in a 20 μl total volume with 2 μl of DNA template, 10 μl GoTaq Hot Start Polymerase (Promega) (25 mM MgCl_2_, GoTaq Hot Start Polymerase, 5× Colourless GoTaq Flexi Buffer, 5× Green GoTaq Flexi Buffer), 1 μM forward primer, 1 μM reverse primer and 6 μl dH_2_O. We amplified a partial sequence of 495 bp of COI, using the primers Cox (5′-TGATTCTTTGGGCATCCTGAAG-3′) and Coy (5′-GGGGTAGTCAGAATAGCGTCG-3′) [42]. Amplification involved 95°C 2 min, 37 cycles of 95°C 30 s, 50°C 45 s, 72°C 45 s, followed by 72°C 5 min (adapted from [43]). For COI amplification protocol of samples from Hume–Whittlesea, refer to Hale *et al.* [37].

We amplified a partial sequence of 665 bp of ND4 for the subset of 112 samples (drawn from samples in all four regions), using the primers ND4-3 (F) (5′-TTAGCAGGAACACTTCTAAAACTAG-3′) and ND4-1 (R) (5′-GAAAGTGTTTAGCTTTCATCTCTAG-3′) [36]. Amplification involved 95°C 2 min, 30 cycles of 95°C 30 s, 50°C 60 s, 72°C 45 s, followed by 72°C 5 min (adapted from [36]). Additionally, we screened four nuclear gene regions: proopiomelanocortin A (POMC), recombinase activating gene 1 (RAG-1), Rhodopsin (Rhod) and β-crystallin (CRYBA1), using a subset of 19 samples that each sequenced a different haplotype at the COI gene region (including two samples from the Hume–Whittlesea region). Protocols are provided in electronic supplementary material, S2.

We diluted extracted DNA to a ratio of 1:10 with dH_2_O in all cases. We routinely included negative controls and checked for contamination. Following successful amplification, we purified PCR products using ExoSAP-IT (USB) following the manufacturer's instructions and sent the purified products to Macrogen (Korea) for sequencing using a 3730XL DNA sequencer (ABI). We BLAST searched all sequences to confirm identity and aligned them using Geneious Pro v. 5.6 [43]. We repeat sequenced all novel haplotypes.

### 2.4 Microsatellite genotyping

We genotyped four polymorphic microsatellite markers (Lr2, Lr6, Lr7 and Lr9) as described by Hale *et al*. [44], in two sets of two marker multiplex PCR amplifications, for a subset of 117 samples (including 27 samples from Hume–Whittlesea). The microsatellite loci chosen for this study had previously been developed and successfully genotyped for *L. raniformis* around Melbourne [44]. Three markers incorporated a GTTTCTT ‘pigtail’ added to the 5^′^ end of the reverse primer to reduce variation in stutter (Lr2, Lr6 and Lr9). See Hale *et al*. [44] for methods for fluorescently labelling fragments for all loci. The PCR was performed in a 10 μl total volume with 1 μl of DNA template (neat), 5 μl GoTaq Hot Start Polymerase (Promega) (25 mM MgCl_2_, GoTaq Hot Start Polymerase, 5× Colourless GoTaq Flexi Buffer, 5× Green GoTaq Flexi Buffer), 0.5 μM reverse primer, 0.15 μM forward primer, 0.25 μM fluorescently labelled 454A primer and 3.1 μl dH_2_O. Amplification involved 95°C 2 min, 42 cycles of 95°C 30 s, 50°C 45 s, 72°C 45 s, followed by 72°C 5 min (adapted from Hale *et al*. [44]). Fragment analysis of PCR products were carried out by Macrogen on an Applied Biosystems ABI3730XL DNA analyser using a LIZ-500 size standard. Scoring was completed using Geneious Pro v. 5.6, and all samples were screened manually for accuracy.

### 2.5 Data analyses

We estimated completeness of haplotype sampling of populations using the Stirling probability distribution and Bayes' theorem [45]. Median-joining haplotype networks were built using the program Network v. 4.610 [46] for COI and ND4 sequence data, for individual gene regions and a concatenated dataset. Haplotype networks were used, as they better illustrate intraspecific genetic divergence when the number of mutations between haplotypes is small [47].

We used DnaSP v. 5.10.1 [48] to detect signatures of a past genetic bottleneck based on mtDNA, estimating: (1) Tajima's test statistic (*D*), where a large, positive value of *D* is consistent with a population that has experienced a recent bottleneck [49], (2) Fu's test statistic (*F*_*S*_), which assesses the number of rare alleles in the population and departures from conditions of neutrality [50], and (3) the raggedness statistic (*r*), which quantifies the smoothness of the observed mismatch distribution and indicates whether populations are expanding or contracting. A substantial mismatch is characteristic of a population not at equilibrium [51]. Values were determined for both COI separately, and then the COI and ND4 concatenated dataset.

For the microsatellite data, we used Micro-Checker (v. 2.2.3) [52] to test for the presence of null alleles, large allele dropout and scoring error due to stuttering. This was calculated across the entire dataset prior to analysing the number of genetic populations present and then for each genetic population individually once these populations had been identified. Expected and observed heterozygosities (*H*_E_ and *H*_O_, respectively) were implemented in Genepop v. 4.2 [53] with default settings, to assess for linkage disequilibrium and deviations from Hardy–Weinberg equilibrium (HWE) across the four microsatellite loci for each population. Significance values were altered for multiple simultaneous tests using the false discovery rate (FDR) correction [54]. We calculated average allelic richness for each population, using HP-Rare v. 1.0, which accounts for differences in sample size [55]. *F*_ST_ between populations was calculated using GenAlEx v. 6.5 [56].

We estimated the number of distinct genetic populations using microsatellites for a subset of 117 samples in the Bayesian population clustering program Structure v. 2.3.4 [57]. We used a correlated frequency model (to increase the power to detect subtle population structure) with admixture (the recommended starting point). The LOCPRIOR model was used, which can incorporate prior information when the signal of structuring is relatively weak [58]. We used geographical region (coded as one of the four regions from which samples were collected, see figure 1) as prior information. Each run had a burn-in of 100 000 Markov chain Monte Carlo samples, with a further 100 000 samples used to characterize population structure. The number of genetic populations (*K*) was set to range from 1 to 12 (where 12 is the number of clusters within regions). Simulations were run 10 times for each proposed value of *K*. We are confident that the chains had converged, as several runs at each *K* with different run lengths gave consistent parameter values. To determine *K*, we used Structure Harvester v. 0.6.93 [59], which plots the mean estimated log probability of the data, log_e_ Pr(*X*|*K*), as well as the rate of change in the log probability of the data between successive *K* estimates. The value of *K* with the highest rate of change and the largest mean log_e_ Pr(*X*|*K*) was selected [60,61]. The alternative inclusion of geographical cluster and COI haplotype as prior information led to ambiguous results with regard to *K* and weak structure that did not seem biologically applicable (e.g. testing *K*=1–12 and a LOCPRIOR of geographical cluster generated *K*=3 or 10, and testing *K*=1–20 and a LOCPRIOR of COI haplotype generated *K*=2; electronic supplementary material, S3).

To detect the signature of a past bottleneck from the microsatellite data, we used Bottleneck v. 1.2.02 [62,63] and *M*-ratio tests calculated in M_P_VAL and Critical_M [64] for each of the two populations. In Bottleneck, we tested for heterozygosity excess, indicative of population size reduction, using a Wilcoxon test applied to a two-phase model, with 78% single step mutations, as recommended by Peery *et al*. [65] and a variance of 12 [63]. An FDR adjustment for multiple comparisons was applied. *M*-ratio tests were calculated in M_P_VAL, using data formatted in FORMATOMATIC v. 0.8.1 [66]. *M*-ratio tests are based on the ratio of the number of microsatellite alleles to the range in allele size. During a bottleneck, the number of alleles is expected to decline faster than the range in allele size, so the *M*-ratio is expected to be smaller in bottlenecked populations [65].

The *M*-ratio method is likely to detect older events than tests based on heterozygosity, because more time is required for the *M*-statistic to reach equilibrium [67]. Three parameters are required for M_P_VAL: theta (*θ*=4*N*_e_*μ*, where *N*_e_=effective population size and *μ*=per generation mutation rate), the average size of mutations that are not one-step and the proportion of multi-step mutations. We calculated *θ* for each population identified by the preceding Structure analysis using Migrate v. 3.6.6 [68]. We employed a Brownian motion model, using four static heating chains (1, 1.5, 3 and 1 000 000), swapping among chains every 10 steps. For each model, we performed 1 000 000 steps, sampled every 100 steps, with a burn-in length of 100 000 steps. The average size of mutations that are not one-step was set as the default 2.8 [64] and 0.22 was used as the recommended proportion of multi-step mutations [65]. Values of *M* were compared with the critical value (*M*_C_), calculated in Critical_M, estimated after 10 000 simulations. In all the analyses using microsatellite data, the low number of markers used could have limited the power of our methods to detect a bottleneck. However, methods based on examining mtDNA haplotypes can be more powerful for detecting bottlenecks than microsatellite methods based on genetic structure [69].

## 3. Results

### 3.1 Genetic diversity

The posterior probabilities of completeness of haplotype sampling, Pr(*n*=*x*), were high for all regions (more than 0.99), indicating a high likelihood that all haplotypes from each region were sampled. At the finer scale, with regions separated into their component site clusters, there was at least 99% certainty of completeness of sampling for all clusters (Pr(*n*=*x*)≥0.99) except the southern-most cluster from the Cardinia region, at which Pr(*n*=*x*)=0.75. Overall, there was a high probability that all COI haplotypes were sampled.

**Figure 2.**
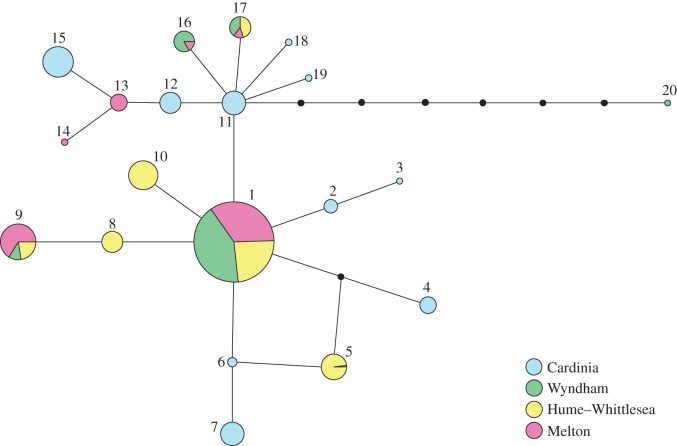
Haplotype network for 495 bp of the COI gene. Each pie represents a unique genetic sequence (haplotype) and the area of the pie is proportional to haplotype frequency within the entire dataset. Each line represents one mutational step. Small black circles correspond to inferred alleles, missing from the dataset.

**Figure 3.**
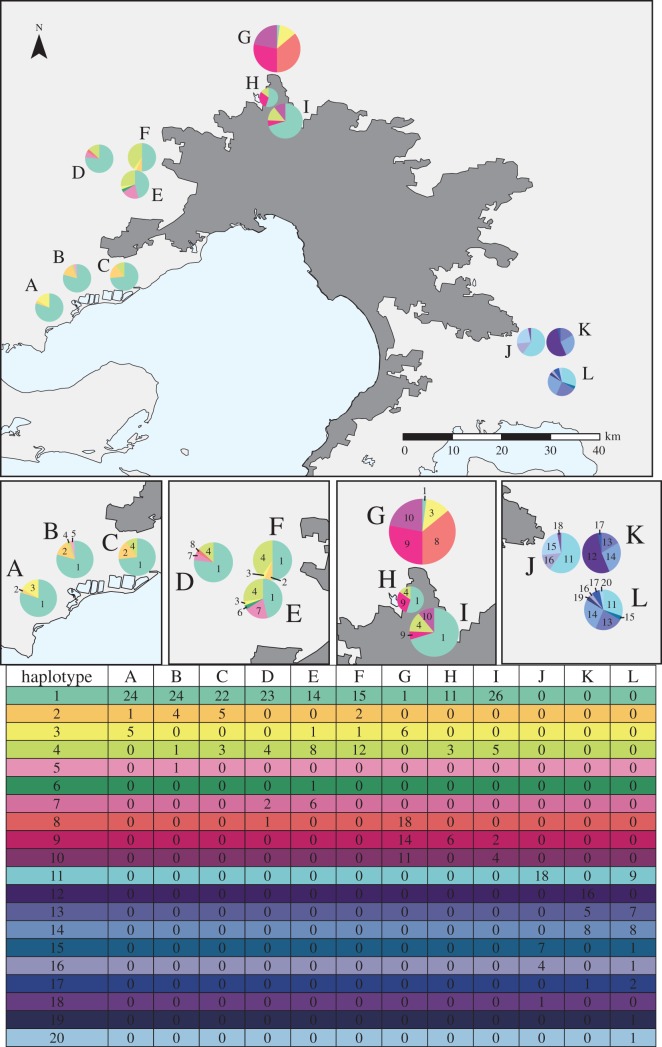
Geographical association of COI haplotypes. Each pie represents the haplotypes found in that cluster and the area of the pie is proportional to sample size. Clusters have been assigned letters and haplotypes numbered. See included table for the number of individuals displaying each haplotype.

A haplotype network was constructed from the 377 aligned COI mitochondrial gene regions (figure 2). In total, there were 22 polymorphic sites, representing 20 distinct haplotypes. Five haplotypes were present in the Wyndham region, seven in Melton, six in Hume–Whittlesea and 10 in Cardinia. There was a common ‘central’ haplotype present (haplotype 1), representing 167 of the 377 samples. All observed haplotypes were a maximum of four mutational steps away from this central haplotype, except one sample from the Wyndham region, which was eight mutational steps away (haplotype 20). One haplotype was unique to the Wyndham region (haplotype 20), two Melton haplotypes were unique (haplotypes 13 and 14), two haplotypes were unique to Hume–Whittlesea (haplotypes 8 and 10) and 10 haplotypes were unique to Cardinia (all the haplotypes observed for the area: haplotypes 2, 3, 4, 6, 7, 11, 12, 15, 18 and 19). Two additional haplotypes (haplotypes 9 and 17) were shared by individuals from Wyndham, Melton and Hume–Whittlesea. One haplotype (haplotype 16) was shared by individuals in Wyndham and Melton, and one haplotype (haplotype 5) was shared by individuals in Melton and Hume–Whittlesea. We also examined the COI haplotype composition of each cluster of sites (figure 3). The central haplotype (haplotype 1) was present in each of the three site clusters from Wyndham, Melton and Hume–Whittlesea (clusters A–I). Patterns of haplotype diversity and exchange between site clusters were also similar in Wyndham, Melton and Hume–Whittlesea.

The haplotype network derived from the 112 aligned ND4 mitochondrial gene regions revealed similar results to those derived from COI (electronic supplementary material, S4). In total, there were 26 distinct haplotypes. Seven haplotypes were present in the Wyndham region (three unique to the region), eight in Melton (three unique), seven in Hume–Whittlesea (five unique) and 11 from the Cardinia region (10 unique). The haplotype network derived from the concatenated COI and ND4 gene regions also revealed similar results to those derived from COI alone (electronic supplementary material, S5). In total, there were 33 distinct haplotypes. Eight haplotypes were present in the Wyndham region (three unique to the region), nine in Melton (three unique), 11 in Hume–Whittlesea (eight unique) and 13 from the Cardinia region (all 13 unique to the region).

Additionally, three nuclear gene regions were aligned, using a subset of the 19 most variable COI samples. The fourth nuclear gene region, CRYBA1, failed to successfully amplify. Rhod and RAG-1 showed no variation between samples. POMC showed variation for eight individuals; however, this variation was not parsimoniously informative, as it occurred at heterozygous sites (refer to electronic supplementary material, S1). Due to the lack of diversity, there was no scope for further population-level analyses with the nuclear DNA.

### 3.2 Population structure

**Figure 4.**
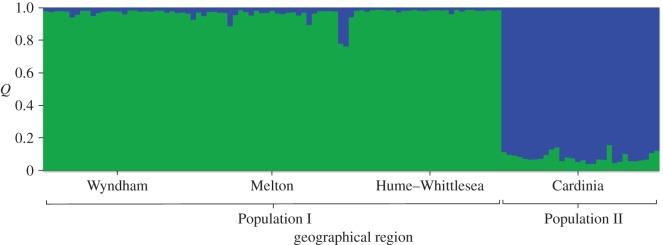
Structure bar plot (*K*=2) with individuals organized by geographical region. Each vertical bar represents a single individual and estimates *Q*: the probability an individual belongs to a population of the given colour.

The Structure analysis using geographical region as prior information revealed two clear genetic units (Δ*K*=34.740 for *K*=2 and Δ*K*=0.024–1.145 for *K*=3–11). Population I contained all samples collected from the Wyndham, Melton and Hume–Whittlesea regions, while Population II contained all samples collected from the Cardinia region in the southeast (figure 4). Each of these populations contained 18 microsatellite alleles. We rejected the possibility of large allele dropout or scoring errors due to stuttering when testing each locus individually. There was no evidence of linkage disequilibrium (*p*=0.025–0.045 following FDR correction for multiple comparisons). Overall, each locus generally conformed to HWE across both populations (table 1). Two loci, Lr6 and Lr9, showed significant deviations from HWE, but the pattern was not consistent across populations (Population I *H*_o_=0.586 for Lr9 and Population II *H*_o_=0.367 for Lr6). We observed significant homozygote excess, suggesting the presence of null alleles in Population II for Lr6; however, we chose to retain all loci for subsequent analyses, given there was no evidence of null alleles in Population I or when analysing the data as one population. Population I had 18 alleles, with an allelic richness of 2.33. Population II had 18 alleles, and an allelic richness of 2.43. Pairwise *F*_ST_ between the two genetic populations was low (0.004) and not statistically significant (*p*=0.176).

**Table 1.** Genetic variability of mtDNA (COI) and microsatellites in populations determined by Structure.

genetic diversity	Population I	Population II
*mtDNA*		
number of samples (*N*)	287	90
length (bp)	495	495
no. haplotypes	10	10
haplotype diversity (*h*)	0.658 (var=0.001, s.d.=0.028)	0.826 (var=0.000, s.d.=0.020)
nucleotide diversity (*π*)	0.003	0.006
average no. nucleotide differences (*k*)	1.463 (obs var=2.009, CV=0.970)	2.950 (obs var=3.835, CV=0.666)
no. polymorphic sites (*S*)	18	10
total no. mutations (*Eta*)	18	11
Tajima's test statistic (*D*)	−1.251 (not stat sig, *p*>0.1)	0.954 (not stat sig, *p*>0.1)
Fu's test statistic (*F*_s_)	−0.853	0.659
raggedness index (*r*)	0.118	0.105
*microsatellites*		
observed heterozygosity (*H*_O_)	0.586	0.550
expected heterozygosity (*H*_E_)	0.608	0.636
allelic richness (AR)	2.330	2.430

### 3.3 Genetic bottleneck tests

There was only weak evidence for a genetic bottleneck based on mtDNA data for COI (table 1). The raggedness statistics showed evidence of weak genetic bottlenecks in both populations; however, neither population deviated significantly from expectations of neutrality, as revealed by Fu's *F*_s_ statistic and Tajima's *D* statistic. No substantial evidence of bottlenecks was found in either population for concatenated regions of COI and ND4 (electronic supplementary material, S6). A one-tailed Wilcoxon's test for heterozygosity excess in Bottleneck found Population I displayed evidence of a recent genetic bottleneck (*p*=0.031); however, this did not remain statistically significant following FDR correction for multiple comparisons (*p*=0.063). Similarly, the *M*-ratio tests on microsatellite loci did not detect evidence of a bottleneck in either of the populations. Using *θ*=3.1 (Population I) and *θ*=0.967 (Population II), the average *M*-ratio values were 0.817–0.888 and were higher than the *M*-critical values, of 0.695–0.697.

## 4. Discussion

This study sought to assess the genetic structure and diversity of remnant populations of *L*. *raniformis* around Melbourne, Australia's fastest growing city. We predicted that: (i) genetic diversity of these populations would be low due to past declines and more recent habitat loss and fragmentation, (ii) genetic bottlenecks would be evident, and (iii) genetic structuring would be present between remnant populations in Cardinia and the other three regions studied.

### 4.1 Patterns of genetic structure and diversity

The mtDNA and nuclear DNA sequences revealed low levels of genetic diversity throughout remnant populations of *L. raniformis* around Melbourne, supporting our first hypothesis. Additionally, a large proportion of individuals included in our study shared one haplotype (haplotype 1), with few mutations between all the haplotypes present. In comparison, a study that looked at COI sequences across 34 amphibian species, from six families and 11 genera, reported an average nucleotide diversity of 0.203 which is considerably higher than the population-level diversity found in our study (0.003 and 0.006, table 1) [70].

Measures of genetic diversity within populations such as allelic richness, haplotype diversity, heterozygosity values and polymorphism have been found to be lower in amphibians in urban areas than in non-urban environments [2,71–75]. It is difficult to make direct comparisons of genetic diversity between species using microsatellites, which is exacerbated by the low number of microsatellites used for this study. Nevertheless, the measures of heterozygosity and allelic richness are broadly similar to those of other anurans, including related taxa in Australia [76]. In a study of the green and golden bell frog (*Litoria aurea*), the sister species to *L. raniformis*, that covered approximately 1000 km, expected heterozygosity levels were reported as high (0.43–0.82, mean 0.69) when compared to other amphibian species [76]. A previous study [37] examined the structure and fragmentation of *L. raniformis* metapopulations in one of our study regions, Hume–Whittlesea. Using data from 11 microsatellite markers, the signature of a recent bottleneck was found at one site. This population was separated from nearby sites by a four-lane highway, which appeared to significantly reduce gene flow. Hale *et al*. [37] suggested that urbanization around Melbourne has the potential to reduce the genetic diversity of *L. raniformis* due to bottlenecks.

We hypothesized that genetic bottlenecks would be evident in the populations of *L. raniformis* studied here, due to both past population declines (resulting from chytridiomycosis in particular) and more recent habitat loss and fragmentation due to urbanization. However, the data did not support this hypothesis, with both mtDNA and nuclear microsatellite loci analyses failing to detect the signature of a bottleneck. For the microsatellite data, we assessed levels of heterozygosity excess, indicative of population size reduction. However, while commonly used, this method is strongly influenced by the more common alleles [77], which may explain why we did not detect a bottleneck. The ability to detect a prior bottleneck is also influenced by the population size pre-bottleneck, the mutational model and parameters chosen [78], statistical power [62,65] the magnitude of the bottleneck and how recently it occurred [78]. Indeed, populations can experience the negative impacts of a bottleneck for many generations before it is detected [65].

One or more of these factors may have influenced our ability to detect a population bottleneck in our study species. However, dramatic declines in the sister species *L. aurea* similarly found no signature of a bottleneck [76]. It seems likely, therefore, that the contemporary pattern of genetic diversity of *L. raniformis* around Melbourne is due to factors other than recent bottlenecks. In the first instance, it may be that remnant populations of this species around Melbourne may not have suffered the drastic reductions in abundance necessary to produce a bottleneck signature [65]. Secondly, the populations studied here may have retained sufficient local connectivity to obscure or prevent bottlenecks [79]. Previous research confirms that *L. raniformis* displays metapopulation dynamics, with occupancy, mark–recapture and genetic data confirming that migration over distances of 1–2 km is crucial to the viability of population networks [30,34,35]. Given their persistence, it may be inferred that the populations sampled during this study continue to exchange migrants with nearby populations, and remain part of a functioning metapopulation. This level of connectivity may have been sufficient to obscure or prevent genetic bottlenecks in the populations studied here. We encourage further research on the ecological and evolutionary drivers of the low genetic diversity but apparent absence of bottlenecks among remnant populations of *L. raniformis* around Melbourne, including more detailed microsatellite analyses.

Genetic structuring was present between remnant populations in Cardinia and the other three regions, due to both the historical and more recent isolation of this region, supporting our third hypothesis. This was demonstrated by both the mtDNA and microsatellite analyses. Of the four main regions in the study, Cardinia exhibited the highest level of genetic diversity and all haplotypes present there were unique to the region. Additionally, the microsatellite analyses revealed Cardinia should be considered a distinct genetic unit from the other regions.

### 4.2 Conservation implications

Our study indicates populations of *L. raniformis* from Cardinia in southeastern Melbourne are genetically distinct from the west and north of Melbourne, due to the presence of unique haplotypes and higher genetic diversity. Under Moritz's [80] definition, which incorporates reciprocal monophyly, the Cardinia region would not qualify as an Evolutionarily Significant Unit (ESU). However, we suggest that populations from this region should be regarded as a separate Management Unit (MU). MUs can be used to address current population structure and short-term management issues, and are considered distinct from ESUs [80]. Based on our results, we recommend that the maintenance of genetic diversity in the Cardinia region be prioritized and that the region should be considered genetically independent of the remainder of urban Melbourne.

The Victorian Government has classified suitable habitat for *L. raniformis* within the urban growth areas into two categories: (1) high-quality habitat that will be protected and managed for the species and (2) habitat of lower conservation significance that can be destroyed for urban development, but for which compensatory habitat is required [33]. Translocation represents a core component of conservation planning for *L. raniformis* in Melbourne's urban growth areas [33]. Individuals occupying habitat designated for destruction (i.e. that defined as category 2 habitat, as above) will be translocated in cases where there are appropriate locations to receive animals, and where risks, including disease, are considered manageable [33]. Under this policy, some of the populations studied here in the Melton, Hume–Whittlesea and Cardinia areas will be translocated during future urban expansion. The appropriateness of amphibian and reptile translocations has been debated widely during the past 20 years and many attempted translocations have been unsuccessful [81]. There is a clear conflict in policies regarding translocations in Victoria. There is currently no evidence that populations of *L. raniformis* can be successfully relocated [32]. Indeed, all translocation attempts for this species and the closely related *L. aurea* have failed or the focal populations are performing poorly [82], despite *L. aurea* being the subject of more translocation attempts than any other Australian frog [83].

If future captive breeding programmes are required or translocations are attempted for *L. raniformis*, it is important that the geographical location of specimens is considered. This will help to maintain the genetic integrity and evolutionary potential of the species around Melbourne, two key objectives of Victoria's Flora and Fauna Guarantee Act 1988. This appears most important in the southeast Cardinia region, to maintain the levels of genetic diversity and the unique haplotypes found in the region. We caution that we only analysed neutral genetic diversity here, when in fact levels of genetic variability inferred from neutral markers are often poor correlates of quantitative variation for adaptively important traits [84]. Nevertheless, we advise that translocation of individuals between regions should be avoided and that fine-scale genetic studies within regions should be undertaken to determine the long-term viability of translocations within regions. By quantifying genetic structure and diversity of *L. raniformis* across Melbourne's urban fringe, using mtDNA and microsatellite markers, we are able to forewarn managers of the low genetic diversity displayed by remnant populations, identify genetic MUs and regional centres of haplotype endemism.

## Supplementary Material

Keely_Supplementary_Material

## Data accessibility

DNA sequences generated in this study have GenBank accessions: KP343052–KP343597.
