# Supplementary material for: Genetic structure and diversity of the endangered growling grass frog in a rapidly urbanizing region
Source: R Soc Open Sci. 2015 Aug 26;2(8):140255. doi: 10.1098/rsos.140255 (PMC4555848; doi:10.1098/rsos.140255)
Supplement: Keely_Supplementary_Material [file rsos140255supp1.docx]

Title: Genetic structure and diversity of the endangered Growling Grass Frog in a rapidly urbanising region

**Authors:** Claire C. Keely, Joshua M. Hale, Geoffrey W. Heard, Kirsten M. Parris, Joanna Sumner, Andrew J. Hamer, Jane Melville

**Corresponding author:** Claire Keely

School of BioSciences, The University of Melbourne, Parkville, VIC 3010, Australia

Phone: +61 3 8344 0120; Fax: +61 3 9347 5460; Email: c.keely@pgrad.unimelb.edu.au

**Supplementary Material 1** Museum Victoria registration numbers, localities of specimens from which DNA was extracted and GenBank accession numbers are provided below.

| Museum ID | Location | Latitude/longitude | COI # | ND4 # | POMC # | RAG-1 # | Rhod # |
| --- | --- | --- | --- | --- | --- | --- | --- |
| NMVZ16504 | Australia:Caroline Springs, Vic | 37.74 S 144.74 E | KP343052 | KP343429 |  |  |  |
| NMVZ16505 | Australia:Caroline Springs, Vic | 37.74 S 144.74 E | KP343053 | KP343430 |  |  |  |
| NMVZ16506 | Australia:Caroline Springs, Vic | 37.74 S 144.75 E | KP343054 |  |  |  |  |
| NMVZ16507 | Australia:Caroline Springs, Vic | 37.74 S 144.75 E | KP343055 |  |  |  |  |
| NMVZ16508 | Australia:Caroline Springs, Vic | 37.74 S 144.75 E | KP343056 |  |  |  |  |
| NMVZ16509 | Australia:Caroline Springs, Vic | 37.74 S 144.75 E | KP343057 |  |  |  |  |
| NMVZ16510 | Australia:Kings Park, Vic | 37.74 S 144.76 E | KP343058 | KP343431 |  |  |  |
| NMVZ16514 | Australia:Kings Park, Vic | 37.74 S 144.76 E | KP343059 |  |  |  |  |
| NMVZ16515 | Australia:Burnside Heights, Vic | 37.74 S 144.76 E | KP343060 |  |  |  |  |
| NMVZ16516 | Australia:Caroline Springs, Vic | 37.74 S 144.74 E | KP343061 | KP343432 |  |  |  |
| NMVZ16517 | Australia:Caroline Springs, Vic | 37.74 S 144.74 E | KP343062 |  |  |  |  |
| NMVZ16518 | Australia:Caroline Springs, Vic | 37.75 S 144.73 E | KP343063 | KP343433 |  |  |  |
| NMVZ16519 | Australia:Caroline Springs, Vic | 37.75 S 144.73 E | KP343064 | KP343434 |  |  |  |
| NMVZ16520 | Australia:Caroline Springs, Vic | 37.75 S 144.73 E | KP343065 |  |  |  |  |
| NMVZ16521 | Australia:Caroline Springs, Vic | 37.75 S 144.73 E | KP343066 | KP343435 | KP343541 | KP343560 | KP343579 |
| NMVZ16522 | Australia:Caroline Springs, Vic | 37.75 S 144.73 E | KP343067 |  |  |  |  |
| NMVZ16526 | Australia:Caroline Springs, Vic | 37.75 S 144.73 E | KP343068 | KP343436 |  |  |  |
| NMVZ16527 | Australia:Caroline Springs, Vic | 37.75 S 144.73 E | KP343069 |  |  |  |  |
| NMVZ16528 | Australia:Caroline Springs, Vic | 37.74 S 144.7 E | KP343070 |  |  |  |  |
| NMVZ16529 | Australia:Caroline Springs, Vic | 37.74 S 144.7 E | KP343071 |  |  |  |  |
| NMVZ16530 | Australia:Caroline Springs, Vic | 37.74 S 144.7 E | KP343072 | KP343437 |  |  |  |
| NMVZ16534 | Australia:Caroline Springs, Vic | 37.74 S 144.74 E | KP343073 | KP343438 |  |  |  |
| NMVZ16535 | Australia:Caroline Springs, Vic | 37.74 S 144.74 E | KP343074 | KP343439 |  |  |  |
| NMVZ16536 | Australia:Caroline Springs, Vic | 37.74 S 144.74 E | KP343075 | KP343440 |  |  |  |
| NMVZ16537 | Australia:Caroline Springs, Vic | 37.74 S 144.74 E | KP343076 |  |  |  |  |
| NMVZ16538 | Australia:Caroline Springs, Vic | 37.74 S 144.74 E | KP343077 |  |  |  |  |
| NMVZ16539 | Australia:Caroline Springs, Vic | 37.74 S 144.75 E | KP343078 |  |  |  |  |
| NMVZ16540 | Australia:Caroline Springs, Vic | 37.74 S 144.75 E | KP343079 |  |  |  |  |
| NMVZ16541 | Australia:Caroline Springs, Vic | 37.74 S 144.75 E | KP343080 |  |  |  |  |
| NMVZ16542 | Australia:Kings Park, Vic | 37.74 S 144.76 E | KP343081 |  |  |  |  |
| NMVZ16543 | Australia:Burnside Heights, Vic | 37.74 S 144.76 E | KP343082 |  |  |  |  |
| NMVZ16544 | Australia:Burnside Heights, Vic | 37.74 S 144.76 E | KP343083 |  |  |  |  |
| NMVZ16548 | Australia:Burnside Heights, Vic | 37.74 S 144.76 E | KP343084 |  |  |  |  |
| NMVZ16549 | Australia:Burnside Heights, Vic | 37.74 S 144.76 E | KP343085 |  |  |  |  |
| NMVZ16550 | Australia:Caroline Springs, Vic | 37.74 S 144.74 E | KP343086 |  |  |  |  |
| NMVZ16551 | Australia:Caroline Springs, Vic | 37.74 S 144.74 E | KP343087 |  |  |  |  |
| NMVZ16552 | Australia:Caroline Springs, Vic | 37.74 S 144.74 E | KP343088 | KP343441 |  |  |  |
| NMVZ16553 | Australia:Caroline Springs, Vic | 37.74 S 144.74 E | KP343089 |  |  |  |  |
| NMVZ16554 | Australia:Caroline Springs, Vic | 37.75 S 144.74 E | KP343090 | KP343442 | KP343542 | KP343561 | KP343580 |
| NMVZ16555 | Australia:Caroline Springs, Vic | 37.75 S 144.74 E | KP343091 |  |  |  |  |
| NMVZ16556 | Australia:Caroline Springs, Vic | 37.75 S 144.74 E | KP343092 |  |  |  |  |
| NMVZ16557 | Australia:Caroline Springs, Vic | 37.75 S 144.74 E | KP343093 | KP343443 | KP343543 | KP343562 | KP343581 |
| NMVZ16558 | Australia:Caroline Springs, Vic | 37.75 S 144.74 E | KP343094 | KP343444 |  |  |  |
| NMVZ16562 | Australia:Caroline Springs, Vic | 37.75 S 144.74 E | KP343095 | KP343445 |  |  |  |
| NMVZ16563 | Australia:Caroline Springs, Vic | 37.75 S 144.74 E | KP343096 |  |  |  |  |
| NMVZ16564 | Australia:Caroline Springs, Vic | 37.75 S 144.74 E | KP343097 |  |  |  |  |
| NMVZ16565 | Australia:Caroline Springs, Vic | 37.75 S 144.74 E | KP343098 |  |  |  |  |
| NMVZ16566 | Australia:Caroline Springs, Vic | 37.75 S 144.74 E | KP343099 |  |  |  |  |
| NMVZ16567 | Australia:Officer, Vic | 38.07 S 145.42 E | KP343100 | KP343446 |  |  |  |
| NMVZ16571 | Australia:Officer, Vic | 38.07 S 145.42 E | KP343101 | KP343447 |  |  |  |
| NMVZ16572 | Australia:Officer, Vic | 38.07 S 145.42 E | KP343102 | KP343448 |  |  |  |
| NMVZ16573 | Australia:Officer, Vic | 38.07 S 145.42 E | KP343103 |  |  |  |  |
| NMVZ16574 | Australia:Officer, Vic | 38.07 S 145.42 E | KP343104 |  |  |  |  |
| NMVZ16575 | Australia:Officer, Vic | 38.07 S 145.42 E | KP343105 |  |  |  |  |
| NMVZ16576 | Australia:Officer, Vic | 38.07 S 145.42 E | KP343106 |  |  |  |  |
| NMVZ16580 | Australia:Caroline Springs, Vic | 37.75 S 144.74 E | KP343107 |  |  |  |  |
| NMVZ16581 | Australia:Caroline Springs, Vic | 37.75 S 144.73 E | KP343108 |  |  |  |  |
| NMVZ16582 | Australia:Caroline Springs, Vic | 37.75 S 144.73 E | KP343109 |  |  |  |  |
| NMVZ16583 | Australia:Caroline Springs, Vic | 37.75 S 144.73 E | KP343110 |  |  |  |  |
| NMVZ16584 | Australia:Caroline Springs, Vic | 37.75 S 144.73 E | KP343111 |  |  |  |  |
| NMVZ16585 | Australia:Caroline Springs, Vic | 37.75 S 144.73 E | KP343112 |  |  |  |  |
| NMVZ16586 | Australia:Caroline Springs, Vic | 37.75 S 144.73 E | KP343113 |  |  |  |  |
| NMVZ16587 | Australia:Caroline Springs, Vic | 37.75 S 144.73 E | KP343114 |  |  |  |  |
| NMVZ16588 | Australia:Caroline Springs, Vic | 37.75 S 144.73 E | KP343115 |  |  |  |  |
| NMVZ16589 | Australia:Caroline Springs, Vic | 37.75 S 144.74 E | KP343116 |  |  |  |  |
| NMVZ16590 | Australia:Caroline Springs, Vic | 37.74 S 144.74 E | KP343117 | KP343449 |  |  |  |
| NMVZ16591 | Australia:Caroline Springs, Vic | 37.74 S 144.74 E | KP343118 |  |  |  |  |
| NMVZ16592 | Australia:Werribee Western Treatment Plant, Vic | 38 S 144.64 E | KP343119 | KP343450 |  |  |  |
| NMVZ16596 | Australia:Werribee Western Treatment Plant, Vic | 38 S 144.64 E | KP343120 |  |  |  |  |
| NMVZ16597 | Australia:Werribee Western Treatment Plant, Vic | 38 S 144.64 E | KP343121 | KP343451 |  |  |  |
| NMVZ16598 | Australia:Werribee Western Treatment Plant, Vic | 38 S 144.64 E | KP343122 | KP343452 |  |  |  |
| NMVZ16599 | Australia:Werribee Western Treatment Plant, Vic | 38 S 144.64 E | KP343123 |  |  |  |  |
| NMVZ16600 | Australia:Werribee Western Treatment Plant, Vic | 38 S 144.64 E | KP343124 |  |  |  |  |
| NMVZ16601 | Australia:Werribee Western Treatment Plant, Vic | 38 S 144.64 E | KP343125 | KP343453 |  |  |  |
| NMVZ16602 | Australia:Werribee Western Treatment Plant, Vic | 38 S 144.64 E | KP343126 | KP343454 |  |  |  |
| NMVZ16603 | Australia:Werribee Western Treatment Plant, Vic | 38 S 144.63 E | KP343127 |  |  |  |  |
| NMVZ17254 | Australia:Werribee Western Treatment Plant, Vic | 38 S 144.63 E | KP343128 |  |  |  |  |
| NMVZ17255 | Australia:Werribee Western Treatment Plant, Vic | 38 S 144.63 E | KP343129 |  |  |  |  |
| NMVZ17259 | Australia:Werribee Western Treatment Plant, Vic | 38 S 144.63 E | KP343130 | KP343455 |  |  |  |
| NMVZ17260 | Australia:Werribee Western Treatment Plant, Vic | 38 S 144.63 E | KP343131 |  |  |  |  |
| NMVZ17261 | Australia:Werribee Western Treatment Plant, Vic | 38 S 144.63 E | KP343132 | KP343456 |  |  |  |
| NMVZ17262 | Australia:Werribee Western Treatment Plant, Vic | 38 S 144.63 E | KP343133 |  |  |  |  |
| NMVZ17266 | Australia:Werribee Western Treatment Plant, Vic | 37.98 S 144.65 E | KP343134 | KP343457 |  |  |  |
| NMVZ17267 | Australia:Werribee Western Treatment Plant, Vic | 37.98 S 144.67 E | KP343135 |  |  |  |  |
| NMVZ17268 | Australia:Werribee Western Treatment Plant, Vic | 37.98 S 144.67 E | KP343136 |  |  |  |  |
| NMVZ17269 | Australia:Werribee Western Treatment Plant, Vic | 37.98 S 144.67 E | KP343137 |  |  |  |  |
| NMVZ17270 | Australia:Werribee Western Treatment Plant, Vic | 37.98 S 144.67 E | KP343138 |  |  |  |  |
| NMVZ17271 | Australia:Werribee Western Treatment Plant, Vic | 37.98 S 144.67 E | KP343139 |  |  |  |  |
| NMVZ17272 | Australia:Werribee Western Treatment Plant, Vic | 37.98 S 144.67 E | KP343140 |  |  |  |  |
| NMVZ17273 | Australia:Werribee Western Treatment Plant, Vic | 37.98 S 144.67 E | KP343141 |  |  |  |  |
| NMVZ17274 | Australia:Werribee Western Treatment Plant, Vic | 37.98 S 144.67 E | KP343142 |  |  |  |  |
| NMVZ17275 | Australia:Werribee Western Treatment Plant, Vic | 37.98 S 144.67 E | KP343143 | KP343458 |  |  |  |
| NMVZ17276 | Australia:Werribee Western Treatment Plant, Vic | 37.98 S 144.67 E | KP343144 |  |  |  |  |
| NMVZ17277 | Australia:Werribee Western Treatment Plant, Vic | 37.98 S 144.67 E | KP343145 |  |  |  |  |
| NMVZ17278 | Australia:Werribee Western Treatment Plant, Vic | 37.98 S 144.67 E | KP343146 |  |  |  |  |
| NMVZ17279 | Australia:Werribee Western Treatment Plant, Vic | 37.98 S 144.67 E | KP343147 |  |  |  |  |
| NMVZ17280 | Australia:Werribee Western Treatment Plant, Vic | 37.98 S 144.67 E | KP343148 |  |  |  |  |
| NMVZ17281 | Australia:Pakenham, Vic | 38.15 S 145.5 E | KP343149 | KP343459 |  |  |  |
| NMVZ17285 | Australia:Pakenham, Vic | 38.15 S 145.5 E | KP343150 | KP343460 |  |  |  |
| NMVZ17286 | Australia:Pakenham, Vic | 38.15 S 145.5 E | KP343151 |  |  |  |  |
| NMVZ17287 | Australia:Pakenham, Vic | 38.15 S 145.5 E | KP343152 | KP343461 | KP343544 | KP343563 | KP343582 |
| NMVZ17288 | Australia:Pakenham, Vic | 38.15 S 145.5 E | KP343153 | KP343462 |  |  |  |
| NMVZ17289 | Australia:Werribee Western Treatment Plant, Vic | 38.04 S 144.51 E | KP343154 |  |  |  |  |
| NMVZ17290 | Australia:Werribee Western Treatment Plant, Vic | 38.04 S 144.51 E | KP343155 | KP343463 |  |  |  |
| NMVZ17291 | Australia:Werribee Western Treatment Plant, Vic | 38.04 S 144.51 E | KP343156 | KP343464 |  |  |  |
| NMVZ17292 | Australia:Werribee Western Treatment Plant, Vic | 38.04 S 144.51 E | KP343157 | KP343465 |  |  |  |
| NMVZ17296 | Australia:Werribee Western Treatment Plant, Vic | 38.04 S 144.51 E | KP343158 | KP343466 | KP343545 | KP343564 | KP343583 |
| NMVZ17297 | Australia:Werribee Western Treatment Plant, Vic | 38.04 S 144.51 E | KP343159 | KP343467 |  |  |  |
| NMVZ17298 | Australia:Werribee Western Treatment Plant, Vic | 38.05 S 144.51 E | KP343160 | KP343468 |  |  |  |
| NMVZ17299 | Australia:Werribee Western Treatment Plant, Vic | 38.05 S 144.51 E | KP343161 |  |  |  |  |
| NMVZ17303 | Australia:Werribee Western Treatment Plant, Vic | 38.05 S 144.51 E | KP343162 | KP343469 |  |  |  |
| NMVZ17304 | Australia:Werribee Western Treatment Plant, Vic | 38.05 S 144.51 E | KP343163 |  |  |  |  |
| NMVZ17305 | Australia:Werribee Western Treatment Plant, Vic | 38.05 S 144.51 E | KP343164 |  |  |  |  |
| NMVZ17306 | Australia:Werribee Western Treatment Plant, Vic | 38.05 S 144.51 E | KP343165 |  |  |  |  |
| NMVZ17307 | Australia:Werribee Western Treatment Plant, Vic | 38.05 S 144.51 E | KP343166 |  |  |  |  |
| NMVZ17308 | Australia:Werribee Western Treatment Plant, Vic | 38.05 S 144.51 E | KP343167 | KP343470 |  |  |  |
| NMVZ17309 | Australia:Werribee Western Treatment Plant, Vic | 38.05 S 144.51 E | KP343168 |  |  |  |  |
| NMVZ17310 | Australia:Werribee Western Treatment Plant, Vic | 38.04 S 144.52 E | KP343169 |  |  |  |  |
| NMVZ17314 | Australia:Werribee Western Treatment Plant, Vic | 38.04 S 144.53 E | KP343170 |  |  |  |  |
| NMVZ17315 | Australia:Werribee Western Treatment Plant, Vic | 38.04 S 144.53 E | KP343171 |  |  |  |  |
| NMVZ17316 | Australia:Werribee Western Treatment Plant, Vic | 38.04 S 144.53 E | KP343172 |  |  |  |  |
| NMVZ17317 | Australia:Werribee Western Treatment Plant, Vic | 38.04 S 144.53 E | KP343173 |  |  |  |  |
| NMVZ17318 | Australia:Werribee Western Treatment Plant, Vic | 38.04 S 144.53 E | KP343174 |  |  |  |  |
| NMVZ17319 | Australia:Werribee Western Treatment Plant, Vic | 38.04 S 144.53 E | KP343175 |  |  |  |  |
| NMVZ17320 | Australia:Werribee Western Treatment Plant, Vic | 38.04 S 144.53 E | KP343176 |  |  |  |  |
| NMVZ17321 | Australia:Werribee Western Treatment Plant, Vic | 38.04 S 144.54 E | KP343177 |  |  |  |  |
| NMVZ17322 | Australia:Werribee Western Treatment Plant, Vic | 38.04 S 144.54 E | KP343178 |  |  |  |  |
| NMVZ17323 | Australia:Werribee Western Treatment Plant, Vic | 38.04 S 144.53 E | KP343179 |  |  |  |  |
| NMVZ17324 | Australia:Werribee Western Treatment Plant, Vic | 38.04 S 144.53 E | KP343180 |  |  |  |  |
| NMVZ17325 | Australia:Werribee Western Treatment Plant, Vic | 38.04 S 144.53 E | KP343181 |  |  |  |  |
| NMVZ17326 | Australia:Werribee Western Treatment Plant, Vic | 38.04 S 144.53 E | KP343182 |  |  |  |  |
| NMVZ17327 | Australia:Werribee Western Treatment Plant, Vic | 38.04 S 144.53 E | KP343183 | KP343471 |  |  |  |
| NMVZ17328 | Australia:Pakenham, Vic | 38.09 S 145.45 E | KP343184 |  |  |  |  |
| NMVZ17332 | Australia:Pakenham, Vic | 38.09 S 145.45 E | KP343185 |  |  |  |  |
| NMVZ17333 | Australia:Pakenham, Vic | 38.09 S 145.45 E | KP343186 |  |  |  |  |
| NMVZ17334 | Australia:Pakenham, Vic | 38.09 S 145.45 E | KP343187 |  |  |  |  |
| NMVZ17335 | Australia:Pakenham, Vic | 38.09 S 145.45 E | KP343188 |  |  |  |  |
| NMVZ17336 | Australia:Pakenham, Vic | 38.09 S 145.45 E | KP343189 |  |  |  |  |
| NMVZ17337 | Australia:Nar Nar Goon, Vic | 38.07 S 145.55 E | KP343190 | KP343472 | KP343546 | KP343565 | KP343584 |
| NMVZ17341 | Australia:Nar Nar Goon, Vic | 38.07 S 145.55 E | KP343191 | KP343473 |  |  |  |
| NMVZ17342 | Australia:Nar Nar Goon, Vic | 38.07 S 145.55 E | KP343192 |  |  |  |  |
| NMVZ17343 | Australia:Pakenham South, Vic | 38.13 S 145.51 E | KP343193 | KP343474 | KP343547 | KP343566 | KP343585 |
| NMVZ17344 | Australia:Pakenham South, Vic | 38.13 S 145.51 E | KP343194 |  |  |  |  |
| NMVZ17345 | Australia:Pakenham South, Vic | 38.13 S 145.51 E | KP343195 |  |  |  |  |
| NMVZ17346 | Australia:Pakenham South, Vic | 38.13 S 145.51 E | KP343196 |  |  |  |  |
| NMVZ17347 | Australia:Pakenham South, Vic | 38.13 S 145.51 E | KP343197 | KP343475 |  |  |  |
| NMVZ17351 | Australia:Nar Nar Goon, Vic | 38.08 S 145.55 E | KP343198 |  |  |  |  |
| NMVZ17352 | Australia:Nar Nar Goon, Vic | 38.08 S 145.55 E | KP343199 | KP343476 |  |  |  |
| NMVZ17353 | Australia:Nar Nar Goon, Vic | 38.08 S 145.55 E | KP343200 | KP343477 |  |  |  |
| NMVZ17354 | Australia:Nar Nar Goon, Vic | 38.08 S 145.55 E | KP343201 |  |  |  |  |
| NMVZ17355 | Australia:Nar Nar Goon, Vic | 38.08 S 145.52 E | KP343202 |  |  |  |  |
| NMVZ17356 | Australia:Nar Nar Goon, Vic | 38.08 S 145.52 E | KP343203 |  |  |  |  |
| NMVZ17357 | Australia:Nar Nar Goon, Vic | 38.08 S 145.52 E | KP343204 |  |  |  |  |
| NMVZ17358 | Australia:Nar Nar Goon, Vic | 38.08 S 145.53 E | KP343205 |  |  |  |  |
| NMVZ17362 | Australia:Nar Nar Goon, Vic | 38.08 S 145.53 E | KP343206 | KP343478 |  |  |  |
| NMVZ17363 | Australia:Nar Nar Goon, Vic | 38.08 S 145.54 E | KP343207 |  |  |  |  |
| NMVZ17364 | Australia:Nar Nar Goon, Vic | 38.07 S 145.55 E | KP343208 |  |  |  |  |
| NMVZ17365 | Australia:Nar Nar Goon, Vic | 38.07 S 145.55 E | KP343209 |  |  |  |  |
| NMVZ17366 | Australia:Nar Nar Goon, Vic | 38.07 S 145.55 E | KP343210 | KP343479 |  |  |  |
| NMVZ17367 | Australia:Nar Nar Goon, Vic | 38.07 S 145.55 E | KP343211 |  |  |  |  |
| NMVZ17368 | Australia:Nar Nar Goon, Vic | 38.07 S 145.55 E | KP343212 |  |  |  |  |
| NMVZ17369 | Australia:Nar Nar Goon, Vic | 38.07 S 145.55 E | KP343213 |  |  |  |  |
| NMVZ17370 | Australia:Nar Nar Goon, Vic | 38.07 S 145.55 E | KP343214 |  |  |  |  |
| NMVZ17371 | Australia:Pakenham, Vic | 38.07 S 145.51 E | KP343215 | KP343480 | KP343548 | KP343567 | KP343586 |
| NMVZ17375 | Australia:Pakenham, Vic | 38.09 S 145.45 E | KP343216 |  |  |  |  |
| NMVZ17376 | Australia:Pakenham, Vic | 38.09 S 145.45 E | KP343217 |  |  |  |  |
| NMVZ17377 | Australia:Pakenham, Vic | 38.09 S 145.45 E | KP343218 |  |  |  |  |
| NMVZ17378 | Australia:Pakenham, Vic | 38.09 S 145.45 E | KP343219 | KP343481 |  |  |  |
| NMVZ17379 | Australia:Pakenham, Vic | 38.09 S 145.45 E | KP343220 |  |  |  |  |
| NMVZ17380 | Australia:Pakenham, Vic | 38.12 S 145.46 E | KP343221 | KP343482 |  |  |  |
| NMVZ17381 | Australia:Pakenham, Vic | 38.12 S 145.46 E | KP343222 | KP343483 |  |  |  |
| NMVZ17382 | Australia:Pakenham, Vic | 38.12 S 145.46 E | KP343223 |  |  |  |  |
| NMVZ17383 | Australia:Pakenham, Vic | 38.12 S 145.46 E | KP343224 | KP343484 | KP343549 | KP343568 | KP343587 |
| NMVZ17384 | Australia:Pakenham, Vic | 38.12 S 145.46 E | KP343225 |  |  |  |  |
| NMVZ17385 | Australia:Rockbank, Vic | 37.69 S 144.67 E | KP343226 | KP343485 | KP343550 | KP343569 | KP343588 |
| NMVZ17389 | Australia:Rockbank, Vic | 37.69 S 144.67 E | KP343227 | KP343486 |  |  |  |
| NMVZ17390 | Australia:Rockbank, Vic | 37.69 S 144.67 E | KP343228 | KP343487 |  |  |  |
| NMVZ17391 | Australia:Rockbank, Vic | 37.69 S 144.67 E | KP343229 | KP343488 |  |  |  |
| NMVZ17392 | Australia:Rockbank, Vic | 37.69 S 144.67 E | KP343230 | KP343489 |  |  |  |
| NMVZ17393 | Australia:Rockbank, Vic | 37.69 S 144.67 E | KP343231 | KP343490 |  |  |  |
| NMVZ17394 | Australia:Rockbank, Vic | 37.69 S 144.67 E | KP343232 |  |  |  |  |
| NMVZ17398 | Australia:Rockbank, Vic | 37.69 S 144.67 E | KP343233 |  |  |  |  |
| NMVZ17399 | Australia:Rockbank, Vic | 37.69 S 144.67 E | KP343234 | KP343491 | KP343551 | KP343570 | KP343589 |
| NMVZ17400 | Australia:Rockbank, Vic | 37.69 S 144.67 E | KP343235 |  |  |  |  |
| NMVZ17401 | Australia:Werribee Western Treatment Plant, Vic | 37.97 S 144.59 E | KP343236 | KP343492 |  |  |  |
| NMVZ17402 | Australia:Werribee Western Treatment Plant, Vic | 37.97 S 144.59 E | KP343237 |  |  |  |  |
| NMVZ17403 | Australia:Werribee Western Treatment Plant, Vic | 37.97 S 144.59 E | KP343238 |  |  |  |  |
| NMVZ17407 | Australia:Werribee Western Treatment Plant, Vic | 37.97 S 144.59 E | KP343239 |  |  |  |  |
| NMVZ17411 | Australia:Werribee Western Treatment Plant, Vic | 37.97 S 144.59 E | KP343240 |  |  |  |  |
| NMVZ17412 | Australia:Werribee Western Treatment Plant, Vic | 37.97 S 144.59 E | KP343241 |  |  |  |  |
| NMVZ17413 | Australia:Werribee Western Treatment Plant, Vic | 37.97 S 144.59 E | KP343242 | KP343493 |  |  |  |
| NMVZ17414 | Australia:Werribee Western Treatment Plant, Vic | 37.97 S 144.59 E | KP343243 |  |  |  |  |
| NMVZ17415 | Australia:Werribee Western Treatment Plant, Vic | 37.97 S 144.59 E | KP343244 | KP343494 |  |  |  |
| NMVZ17416 | Australia:Werribee Western Treatment Plant, Vic | 37.97 S 144.59 E | KP343245 |  |  |  |  |
| NMVZ17417 | Australia:Werribee Western Treatment Plant, Vic | 37.97 S 144.56 E | KP343246 |  |  |  |  |
| NMVZ17418 | Australia:Cardinia, Vic | 38.09 S 145.43 E | KP343247 | KP343495 |  |  |  |
| NMVZ17419 | Australia:Cardinia, Vic | 38.09 S 145.43 E | KP343248 | KP343496 |  |  |  |
| NMVZ17420 | Australia:Pakenham, Vic | 38.09 S 145.46 E | KP343249 |  |  |  |  |
| NMVZ17421 | Australia:Pakenham, Vic | 38.07 S 145.53 E | KP343250 | KP343497 |  |  |  |
| NMVZ17422 | Australia:Nar Nar Goon, Vic | 38.07 S 145.53 E | KP343251 | KP343498 |  |  |  |
| NMVZ17423 | Australia:Nar Nar Goon, Vic | 38.07 S 145.53 E | KP343252 |  |  |  |  |
| NMVZ17424 | Australia:Nar Nar Goon, Vic | 38.07 S 145.53 E | KP343253 |  |  |  |  |
| NMVZ17425 | Australia:Nar Nar Goon, Vic | 38.07 S 145.53 E | KP343254 |  |  |  |  |
| NMVZ17426 | Australia:Nar Nar Goon, Vic | 38.07 S 145.53 E | KP343255 |  |  |  |  |
| NMVZ17427 | Australia:Nar Nar Goon, Vic | 38.07 S 145.53 E | KP343256 |  |  |  |  |
| NMVZ17428 | Australia:Werribee Western Treatment Plant, Vic | 37.97 S 144.62 E | KP343257 |  |  |  |  |
| NMVZ17429 | Australia:Werribee Western Treatment Plant, Vic | 37.97 S 144.62 E | KP343258 |  |  |  |  |
| NMVZ17430 | Australia:Werribee Western Treatment Plant, Vic | 37.97 S 144.62 E | KP343259 |  |  |  |  |
| NMVZ17434 | Australia:Werribee Western Treatment Plant, Vic | 37.97 S 144.62 E | KP343260 |  |  |  |  |
| NMVZ17435 | Australia:Werribee Western Treatment Plant, Vic | 37.97 S 144.62 E | KP343261 |  |  |  |  |
| NMVZ17436 | Australia:Werribee Western Treatment Plant, Vic | 37.97 S 144.62 E | KP343262 |  |  |  |  |
| NMVZ17437 | Australia:Werribee Western Treatment Plant, Vic | 37.98 S 144.57 E | KP343263 | KP343499 |  |  |  |
| NMVZ17441 | Australia:Werribee Western Treatment Plant, Vic | 37.98 S 144.57 E | KP343264 | KP343500 |  |  |  |
| NMVZ17442 | Australia:Werribee Western Treatment Plant, Vic | 37.98 S 144.57 E | KP343265 |  |  |  |  |
| NMVZ17443 | Australia:Werribee Western Treatment Plant, Vic | 38 S 144.55 E | KP343266 |  |  |  |  |
| NMVZ17444 | Australia:Werribee Western Treatment Plant, Vic | 38 S 144.55 E | KP343267 |  |  |  |  |
| NMVZ17445 | Australia:Werribee Western Treatment Plant, Vic | 37.98 S 144.55 E | KP343268 |  |  |  |  |
| NMVZ17446 | Australia:Werribee Western Treatment Plant, Vic | 37.98 S 144.57 E | KP343269 |  |  |  |  |
| NMVZ17447 | Australia:Werribee Western Treatment Plant, Vic | 37.98 S 144.57 E | KP343270 |  |  |  |  |
| NMVZ17448 | Australia:Plumpton, Vic | 37.7 S 144.66 E | KP343271 |  |  |  |  |
| NMVZ17449 | Australia:Plumpton, Vic | 37.7 S 144.66 E | KP343272 |  |  |  |  |
| NMVZ17450 | Australia:Plumpton, Vic | 37.7 S 144.66 E | KP343273 |  |  |  |  |
| NMVZ17451 | Australia:Plumpton, Vic | 37.7 S 144.66 E | KP343274 |  |  |  |  |
| NMVZ17452 | Australia:Plumpton, Vic | 37.7 S 144.66 E | KP343275 |  |  |  |  |
| NMVZ17453 | Australia:Plumpton, Vic | 37.7 S 144.66 E | KP343276 |  |  |  |  |
| NMVZ17457 | Australia:Rockbank, Vic | 37.7 S 144.63 E | KP343277 | KP343501 |  |  |  |
| NMVZ17461 | Australia:Rockbank, Vic | 37.7 S 144.63 E | KP343278 |  |  |  |  |
| NMVZ17462 | Australia:Rockbank, Vic | 37.7 S 144.63 E | KP343279 |  |  |  |  |
| NMVZ17463 | Australia:Rockbank, Vic | 37.7 S 144.63 E | KP343280 | KP343502 |  |  |  |
| NMVZ17464 | Australia:Rockbank, Vic | 37.7 S 144.63 E | KP343281 |  |  |  |  |
| NMVZ17465 | Australia:Pakenham, Vic | 38.1 S 145.46 E | KP343282 |  |  |  |  |
| NMVZ17466 | Australia:Pakenham, Vic | 38.1 S 145.46 E | KP343283 |  |  |  |  |
| NMVZ17467 | Australia:Pakenham, Vic | 38.1 S 145.46 E | KP343284 |  |  |  |  |
| NMVZ17468 | Australia:Pakenham South, Vic | 38.14 S 145.53 E | KP343285 |  |  |  |  |
| NMVZ17469 | Australia:Pakenham South, Vic | 38.14 S 145.53 E | KP343286 |  |  |  |  |
| NMVZ17473 | Australia:Nar Nar Goon, Vic | 38.12 S 145.54 E | KP343287 |  |  |  |  |
| NMVZ17474 | Australia:Nar Nar Goon, Vic | 38.12 S 145.54 E | KP343288 |  |  |  |  |
| NMVZ17475 | Australia:Bayles, Vic | 38.16 S 145.54 E | KP343289 |  |  |  |  |
| NMVZ17476 | Australia:Bayles, Vic | 38.16 S 145.54 E | KP343290 |  |  |  |  |
| NMVZ17477 | Australia:Bayles, Vic | 38.16 S 145.54 E | KP343291 |  |  |  |  |
| NMVZ17478 | Australia:Bayles, Vic | 38.16 S 145.54 E | KP343292 |  |  |  |  |
| NMVZ17479 | Australia:Bayles, Vic | 38.16 S 145.54 E | KP343293 | KP343503 | KP343552 | KP343571 | KP343590 |
| NMVZ17480 | Australia:Bayles, Vic | 38.16 S 145.54 E | KP343294 |  |  |  |  |
| NMVZ17484 | Australia:Bayles, Vic | 38.16 S 145.54 E | KP343295 | KP343504 | KP343553 | KP343572 | KP343591 |
| NMVZ17485 | Australia:Pakenham, Vic | 38.09 S 145.45 E | KP343296 |  |  |  |  |
| NMVZ17486 | Australia:Nar Nar Goon, Vic | 38.13 S 145.56 E | KP343297 | KP343505 | KP343554 | KP343573 | KP343592 |
| NMVZ17487 | Australia:Nar Nar Goon, Vic | 38.13 S 145.56 E | KP343298 | KP343506 | KP343555 | KP343574 | KP343593 |
| NMVZ17488 | Australia:Nar Nar Goon, Vic | 38.13 S 145.56 E | KP343299 | KP343507 |  |  |  |
| NMVZ17489 | Australia:Nar Nar Goon, Vic | 38.13 S 145.56 E | KP343300 |  |  |  |  |
| NMVZ17490 | Australia:Nar Nar Goon, Vic | 38.13 S 145.56 E | KP343301 |  |  |  |  |
| NMVZ17491 | Australia:Werribee Western Treatment Plant, Vic | 37.99 S 144.57 E | KP343302 | KP343508 |  |  |  |
| NMVZ17492 | Australia:Werribee Western Treatment Plant, Vic | 37.99 S 144.57 E | KP343303 | KP343509 | KP343556 | KP343575 | KP343594 |
| NMVZ17493 | Australia:Werribee Western Treatment Plant, Vic | 37.99 S 144.57 E | KP343304 | KP343510 |  |  |  |
| NMVZ17494 | Australia:Werribee Western Treatment Plant, Vic | 37.99 S 144.57 E | KP343305 | KP343511 |  |  |  |
| NMVZ17495 | Australia:Werribee Western Treatment Plant, Vic | 37.99 S 144.57 E | KP343306 | KP343512 |  |  |  |
| NMVZ17496 | Australia:Koo Wee Rup North, Vic | 38.15 S 145.55 E | KP343307 | KP343513 | KP343557 | KP343576 | KP343595 |
| NMVZ17497 | Australia:Koo Wee Rup North, Vic | 38.15 S 145.55 E | KP343308 |  |  |  |  |
| NMVZ17498 | Australia:Koo Wee Rup North, Vic | 38.15 S 145.55 E | KP343309 |  |  |  |  |
| NMVZ17499 | Australia:Koo Wee Rup North, Vic | 38.15 S 145.55 E | KP343310 |  |  |  |  |
| NMVZ17500 | Australia:Nar Nar Goon, Vic | 38.08 S 145.53 E | KP343311 |  |  |  |  |
| NMVZ17501 | Australia:Nar Nar Goon, Vic | 38.08 S 145.53 E | KP343312 |  |  |  |  |
| NMVZ17502 | Australia:Rockbank, Vic | 37.73 S 144.67 E | KP343313 |  |  |  |  |
| NMVZ17506 | Australia:Rockbank, Vic | 37.73 S 144.67 E | KP343314 |  |  |  |  |
| NMVZ17507 | Australia:Rockbank, Vic | 37.73 S 144.67 E | KP343315 |  |  |  |  |
| NMVZ17508 | Australia:Rockbank, Vic | 37.73 S 144.67 E | KP343316 |  |  |  |  |
| NMVZ17509 | Australia:Rockbank, Vic | 37.73 S 144.67 E | KP343317 |  |  |  |  |
| NMVZ17510 | Australia:Rockbank, Vic | 37.73 S 144.67 E | KP343318 |  |  |  |  |
| NMVZ17511 | Australia:Rockbank, Vic | 37.73 S 144.67 E | KP343319 |  |  |  |  |
| NMVZ17512 | Australia:Rockbank, Vic | 37.73 S 144.67 E | KP343320 |  |  |  |  |
| NMVZ17513 | Australia:Rockbank, Vic | 37.73 S 144.67 E | KP343321 |  |  |  |  |
| NMVZ31401 | Australia:Campbellfield, Vic | 37.69 S 144.96 E | KP343322 |  |  |  |  |
| NMVZ31400 | Australia:Campbellfield, Vic | 37.69 S 144.96 E | KP343323 |  |  |  |  |
| NMVZ31399 | Australia:Campbellfield, Vic | 37.69 S 144.96 E | KP343324 |  |  |  |  |
| NMVZ31397 | Australia:Campbellfield, Vic | 37.69 S 144.96 E | KP343325 |  |  |  |  |
| NMVZ31396 | Australia:Campbellfield, Vic | 37.69 S 144.96 E | KP343326 |  |  |  |  |
| NMVZ31393 | Australia:Campbellfield, Vic | 37.69 S 144.96 E | KP343327 |  |  |  |  |
| NMVZ30952 | Australia:Campbellfield, Vic | 37.69 S 144.96 E | KP343328 |  |  |  |  |
| NMVZ31398 | Australia:Campbellfield, Vic | 37.69 S 144.96 E | KP343329 |  |  |  |  |
| NMVZ31394 | Australia:Campbellfield, Vic | 37.69 S 144.96 E | KP343330 | KP343514 |  |  |  |
| NMVZ31391 | Australia:Campbellfield, Vic | 37.69 S 144.96 E | KP343331 | KP343515 |  |  |  |
| NMVZ31384 | Australia:Campbellfield, Vic | 37.69 S 144.98 E | KP343332 |  |  |  |  |
| NMVZ31386 | Australia:Campbellfield, Vic | 37.69 S 144.98 E | KP343333 | KP343516 |  |  |  |
| NMVZ31385 | Australia:Campbellfield, Vic | 37.69 S 144.98 E | KP343334 | KP343517 |  |  |  |
| NMVZ31368 | Australia:Campbellfield, Vic | 37.69 S 144.98 E | KP343335 | KP343518 |  |  |  |
| NMVZ31371 | Australia:Campbellfield, Vic | 37.69 S 144.98 E | KP343336 |  |  |  |  |
| NMVZ31381 | Australia:Campbellfield, Vic | 37.69 S 144.98 E | KP343337 |  |  |  |  |
| NMVZ31363 | Australia:Campbellfield, Vic | 37.69 S 144.98 E | KP343338 | KP343519 |  |  |  |
| NMVZ31365 | Australia:Campbellfield, Vic | 37.69 S 144.98 E | KP343339 | KP343520 |  |  |  |
| NMVZ31370 | Australia:Campbellfield, Vic | 37.69 S 144.98 E | KP343340 | KP343521 |  |  |  |
| NMVZ31358 | Australia:Campbellfield, Vic | 37.69 S 144.98 E | KP343341 | KP343522 |  |  |  |
| NMVZ31500 | Australia:Somerton, Vic | 37.63 S 144.96 E | KP343342 |  |  |  |  |
| NMVZ31516 | Australia:Somerton, Vic | 37.63 S 144.96 E | KP343343 |  |  |  |  |
| NMVZ31513 | Australia:Somerton, Vic | 37.63 S 144.96 E | KP343344 |  |  |  |  |
| NMVZ31503 | Australia:Somerton, Vic | 37.63 S 144.96 E | KP343345 |  |  |  |  |
| NMVZ31498 | Australia:Somerton, Vic | 37.63 S 144.96 E | KP343346 |  |  |  |  |
| NMVZ31489 | Australia:Somerton, Vic | 37.63 S 144.96 E | KP343347 |  |  |  |  |
| NMVZ31499 | Australia:Somerton, Vic | 37.63 S 144.96 E | KP343348 |  |  |  |  |
| NMVZ31515 | Australia:Somerton, Vic | 37.63 S 144.96 E | KP343349 |  |  |  |  |
| NMVZ31509 | Australia:Somerton, Vic | 37.63 S 144.96 E | KP343350 |  |  |  |  |
| NMVZ31492 | Australia:Somerton, Vic | 37.63 S 144.96 E | KP343351 |  |  |  |  |
| NMVZ31447 | Australia:Somerton, Vic | 37.63 S 144.96 E | KP343352 |  |  |  |  |
| NMVZ31474 | Australia:Somerton, Vic | 37.63 S 144.96 E | KP343353 | KP343523 |  |  |  |
| NMVZ31473 | Australia:Somerton, Vic | 37.63 S 144.96 E | KP343354 | KP343524 |  |  |  |
| NMVZ31469 | Australia:Somerton, Vic | 37.63 S 144.96 E | KP343355 |  |  |  |  |
| NMVZ31437 | Australia:Somerton, Vic | 37.63 S 144.97 E | KP343356 |  |  |  |  |
| NMVZ31446 | Australia:Somerton, Vic | 37.63 S 144.97 E | KP343357 |  |  |  |  |
| NMVZ31463 | Australia:Somerton, Vic | 37.63 S 144.97 E | KP343358 |  |  |  |  |
| NMVZ31442 | Australia:Somerton, Vic | 37.64 S 144.97 E | KP343359 |  |  |  |  |
| NMVZ31444 | Australia:Somerton, Vic | 37.64 S 144.97 E | KP343360 |  |  |  |  |
| NMVZ31452 | Australia:Somerton, Vic | 37.64 S 144.97 E | KP343361 | KP343525 |  |  |  |
| NMVZ31445 | Australia:Somerton, Vic | 37.64 S 144.97 E | KP343362 |  |  |  |  |
| NMVZ31453 | Australia:Somerton, Vic | 37.64 S 144.97 E | KP343363 |  |  |  |  |
| NMVZ31448 | Australia:Somerton, Vic | 37.64 S 144.97 E | KP343364 |  |  |  |  |
| NMVZ31434 | Australia:Somerton, Vic | 37.64 S 144.97 E | KP343365 |  |  |  |  |
| NMVZ31439 | Australia:Somerton, Vic | 37.64 S 144.97 E | KP343366 |  |  |  |  |
| NMVZ31467 | Australia:Somerton, Vic | 37.64 S 144.97 E | KP343367 |  |  |  |  |
| NMVZ31468 | Australia:Somerton, Vic | 37.64 S 144.97 E | KP343368 |  |  |  |  |
| NMVZ31485 | Australia:Somerton, Vic | 37.64 S 144.97 E | KP343369 |  |  |  |  |
| NMVZ31490 | Australia:Somerton, Vic | 37.64 S 144.97 E | KP343370 |  |  |  |  |
| NMVZ31497 | Australia:Somerton, Vic | 37.64 S 144.97 E | KP343371 | KP343526 |  |  |  |
| NMVZ31495 | Australia:Somerton, Vic | 37.64 S 144.97 E | KP343372 |  |  |  |  |
| NMVZ31505 | Australia:Somerton, Vic | 37.64 S 144.97 E | KP343373 | KP343527 |  |  |  |
| NMVZ31476 | Australia:Somerton, Vic | 37.64 S 144.97 E | KP343374 |  |  |  |  |
| NMVZ31502 | Australia:Somerton, Vic | 37.64 S 144.97 E | KP343375 | KP343528 |  |  |  |
| NMVZ31496 | Australia:Somerton, Vic | 37.64 S 144.97 E | KP343376 | KP343529 |  |  |  |
| NMVZ31487 | Australia:Somerton, Vic | 37.64 S 144.97 E | KP343377 | KP343530 |  |  |  |
| NMVZ31477 | Australia:Somerton, Vic | 37.64 S 144.97 E | KP343378 | KP343531 |  |  |  |
| NMVZ31432 | Australia:Donnybrook, Vic | 37.54 S 144.97 E | KP343379 |  |  |  |  |
| NMVZ31416 | Australia:Donnybrook, Vic | 37.54 S 144.97 E | KP343380 |  |  |  |  |
| NMVZ31403 | Australia:Donnybrook, Vic | 37.54 S 144.97 E | KP343381 |  |  |  |  |
| NMVZ31424 | Australia:Donnybrook, Vic | 37.54 S 144.97 E | KP343382 |  |  |  |  |
| NMVZ31420 | Australia:Donnybrook, Vic | 37.54 S 144.97 E | KP343383 |  |  |  |  |
| NMVZ31428 | Australia:Donnybrook, Vic | 37.54 S 144.97 E | KP343384 |  |  |  |  |
| NMVZ31430 | Australia:Donnybrook, Vic | 37.54 S 144.97 E | KP343385 |  |  |  |  |
| NMVZ31405 | Australia:Donnybrook, Vic | 37.54 S 144.97 E | KP343386 |  |  |  |  |
| NMVZ31433 | Australia:Donnybrook, Vic | 37.54 S 144.97 E | KP343387 |  |  |  |  |
| NMVZ31419 | Australia:Donnybrook, Vic | 37.54 S 144.97 E | KP343388 |  |  |  |  |
| NMVZ31418 | Australia:Donnybrook, Vic | 37.54 S 144.96 E | KP343389 |  |  |  |  |
| NMVZ31422 | Australia:Donnybrook, Vic | 37.54 S 144.96 E | KP343390 |  |  |  |  |
| NMVZ31408 | Australia:Donnybrook, Vic | 37.54 S 144.96 E | KP343391 |  |  |  |  |
| NMVZ31426 | Australia:Donnybrook, Vic | 37.54 S 144.96 E | KP343392 |  |  |  |  |
| NMVZ31409 | Australia:Donnybrook, Vic | 37.54 S 144.96 E | KP343393 |  |  |  |  |
| NMVZ31412 | Australia:Donnybrook, Vic | 37.54 S 144.96 E | KP343394 |  |  |  |  |
| NMVZ31415 | Australia:Donnybrook, Vic | 37.54 S 144.96 E | KP343395 |  |  |  |  |
| NMVZ31414 | Australia:Donnybrook, Vic | 37.54 S 144.96 E | KP343396 |  |  |  |  |
| NMVZ31421 | Australia:Donnybrook, Vic | 37.54 S 144.96 E | KP343397 |  |  |  |  |
| NMVZ31410 | Australia:Donnybrook, Vic | 37.54 S 144.96 E | KP343398 |  |  |  |  |
| NMVZ31546 | Australia:Donnybrook, Vic | 37.54 S 144.96 E | KP343399 |  |  |  |  |
| NMVZ31562 | Australia:Donnybrook, Vic | 37.54 S 144.96 E | KP343400 |  |  |  |  |
| NMVZ31549 | Australia:Donnybrook, Vic | 37.54 S 144.96 E | KP343401 |  |  |  |  |
| NMVZ31560 | Australia:Donnybrook, Vic | 37.54 S 144.96 E | KP343402 | KP343532 |  |  |  |
| NMVZ30870 | Australia:Donnybrook, Vic | 37.54 S 144.96 E | KP343403 |  |  |  |  |
| NMVZ31574 | Australia:Donnybrook, Vic | 37.54 S 144.96 E | KP343404 |  |  |  |  |
| NMVZ31533 | Australia:Donnybrook, Vic | 37.54 S 144.96 E | KP343405 |  |  |  |  |
| NMVZ31564 | Australia:Donnybrook, Vic | 37.54 S 144.96 E | KP343406 |  |  |  |  |
| NMVZ31561 | Australia:Donnybrook, Vic | 37.54 S 144.96 E | KP343407 |  |  |  |  |
| NMVZ31556 | Australia:Donnybrook, Vic | 37.54 S 144.96 E | KP343408 |  |  |  |  |
| NMVZ31548 | Australia:Donnybrook, Vic | 37.54 S 144.95 E | KP343409 |  |  |  |  |
| NMVZ31539 | Australia:Donnybrook, Vic | 37.54 S 144.95 E | KP343410 |  |  |  |  |
| NMVZ31571 | Australia:Donnybrook, Vic | 37.54 S 144.95 E | KP343411 |  |  |  |  |
| NMVZ31534 | Australia:Donnybrook, Vic | 37.54 S 144.95 E | KP343412 |  |  |  |  |
| NMVZ31545 | Australia:Donnybrook, Vic | 37.54 S 144.95 E | KP343413 |  |  |  |  |
| NMVZ31537 | Australia:Donnybrook, Vic | 37.54 S 144.95 E | KP343414 |  |  |  |  |
| NMVZ31554 | Australia:Donnybrook, Vic | 37.54 S 144.95 E | KP343415 | KP343533 |  |  |  |
| NMVZ31544 | Australia:Donnybrook, Vic | 37.54 S 144.95 E | KP343416 | KP343534 |  |  |  |
| NMVZ31535 | Australia:Donnybrook, Vic | 37.54 S 144.95 E | KP343417 | KP343535 |  |  |  |
| NMVZ31559 | Australia:Donnybrook, Vic | 37.54 S 144.95 E | KP343418 | KP343536 | KP343558 | KP343577 | KP343596 |
| NMVZ31351 | Australia:Donnybrook, Vic | 37.55 S 144.94 E | KP343419 |  |  |  |  |
| NMVZ31355 | Australia:Donnybrook, Vic | 37.55 S 144.94 E | KP343420 |  |  |  |  |
| NMVZ31339 | Australia:Donnybrook, Vic | 37.55 S 144.94 E | KP343421 |  |  |  |  |
| NMVZ31357 | Australia:Donnybrook, Vic | 37.55 S 144.94 E | KP343422 |  |  |  |  |
| NMVZ31342 | Australia:Donnybrook, Vic | 37.55 S 144.94 E | KP343423 |  |  |  |  |
| NMVZ31343 | Australia:Donnybrook, Vic | 37.55 S 144.94 E | KP343424 |  |  |  |  |
| NMVZ31340 | Australia:Donnybrook, Vic | 37.55 S 144.94 E | KP343425 | KP343537 |  |  |  |
| NMVZ31356 | Australia:Donnybrook, Vic | 37.55 S 144.94 E | KP343426 | KP343538 |  |  |  |
| NMVZ31338 | Australia:Donnybrook, Vic | 37.55 S 144.94 E | KP343427 | KP343539 |  |  |  |
| NMVZ31352 | Australia:Donnybrook, Vic | 37.55 S 144.94 E | KP343428 | KP343540 | KP343559 | KP343578 | KP343597 |

**Supplementary Material 2**

We sequenced four nuclear gene regions using a subset of the 19 most variable samples at the COI gene region. For the nuclear gene proopiomelanocortin A (POMC), we amplified a 611 bp sequence, using the primers POMC-1 (5’-GAATGTATYAAAGMMTGCAAGATGGWCCT-3’) and POMC-2 (5’-TAYTGRCCCTTYTTGTGGGCRTT-3’) ([Wiens et al. 2005](#_ENREF_5)). Amplification involved 95°C 2 min, 40 cycles of 95°C 30 s, 50°C 45 s, 72°C 45 s, followed by 72°C 5 min.

For the nuclear gene recombinase activating gene 1 (RAG-1), we amplified a 814 bp sequence, using the primers RS1f (F) (5’-TGCAGTCAGTAYCAYAARATGTAC-3’) P. Chippindale (*pers com*) from ([Gomez-Mestre et al. 2008](#_ENREF_4)) and R1-GFR (5’-GAAGCGCCTGAACAGTTTATTAC-3’) ([Faivovich et al. 2005](#_ENREF_3)). Amplification involved 95°C 2 min, 40 cycles of 95°C 30 s, 51.9°C 45 s, 72°C 45 s, followed by 72°C 5 min

For the nuclear gene Rhodopsin (Rhod), we amplified a 321 bp sequence, using the primers Rhod 1A (5’- ACCATGAACGGAACAGAAGGYCC-3’) and Rhod 1C (5’- CCAAGGGTAGCGAAGAARCCTTC-3’) ([Bossuyt and Milinkovitch 2000](#_ENREF_1)). Amplification involved 95°C 2 min, 40 cycles of 95°C 30 s, 60°C 45 s, 72°C 45 s, followed by 72°C 5 min.

For the nuclear gene β-crystallin (CRYBA1), we were unsuccessful in amplifying a partial gene region, using the primers CRYB1Ls (5’-CGCCTGATGTCTTTCCGCC-3’) and CRYB2Ls (5’-CCAATGAAGTTCTCTTTCTCAA-3’) ([Dolman and Phillips 2004](#_ENREF_2)).

References

Bossuyt, F. and M. C. Milinkovitch. 2000. Convergent adaptive radiations in Madagascan and Asian ranid frogs reveal covariation between larval and adult traits. Proceedings of the National Academy of Sciences **97**:6585-6590.

Dolman, G. and B. Phillips. 2004. Single copy nuclear DNA markers characterized for comparative phylogeography in Australian wet tropics rainforest skinks. Molecular Ecology Notes **4**:185-187.

Faivovich, J., C. F. B. Haddad, P. C. A. Garcia, D. R. Frost, J. A. Campbell, and W. C. Wheeler. 2005. Systematic review of the frog family Hylidae, with special reference to Hylinae: phylogenetic analysis and taxonomic revision. Bulletin of the American Museum of Natural History **294**:1-240.

Gomez-Mestre, I., J. J. Wiens, and K. M. Warkentin. 2008. Evolution of adaptive plasticity: risk-sensitive hatching in neotropical leaf-breeding treefrogs. Ecological Monographs **78**:205-224.

Wiens, J. J., J. W. Fetzner Jr, C. L. Parkinson, and T. W. Reeder. 2005. Hylid frog phylogeny and sampling strategies for speciose clades. Systematic Biology **54**:778-807.

**Supplementary Material 3**

We tested geographic cluster and COI haplotype as prior information in our initial Structure analyses. The resulting output was ambiguous with regard to *K* and indicated weak population structure that did not seem biologically likely. As a result these Structure results were not included in the manuscript, but are recorded below. All other parameters are the same as in the manuscript methods.

1. Structure results using 20 COI haplotype groups as prior information. Line graph of Delta *K* indicates 2 populations most likely (*K*=2 is the highest value). The 2 populations are represented as green and blue in the bar plot, which has been ordered into the 20 haplotype groups.


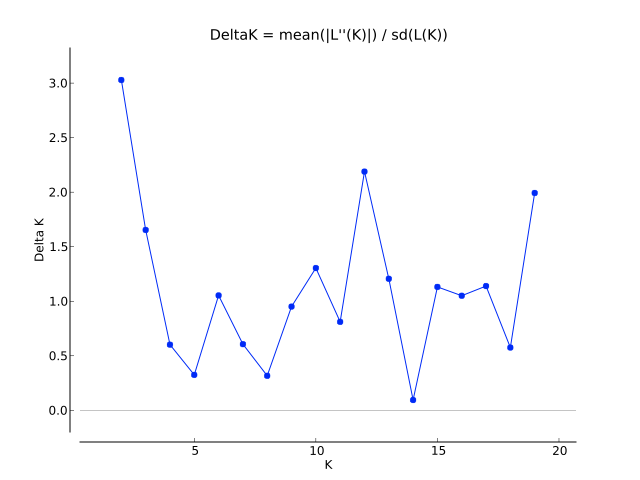


2. Structure results using 12 geographic clusters as prior information. Line graph of Delta *K* indicates 3 or 10 populations most likely (*K*=3 and *K*=10 are the highest values). These populations are represented as different colours in the bar plots below. These bar plots have been ordered into the 12 geographic clusters, as indicated by labels A-L (refer to Figure 3 of manuscript for location of clusters A-L around Melbourne).


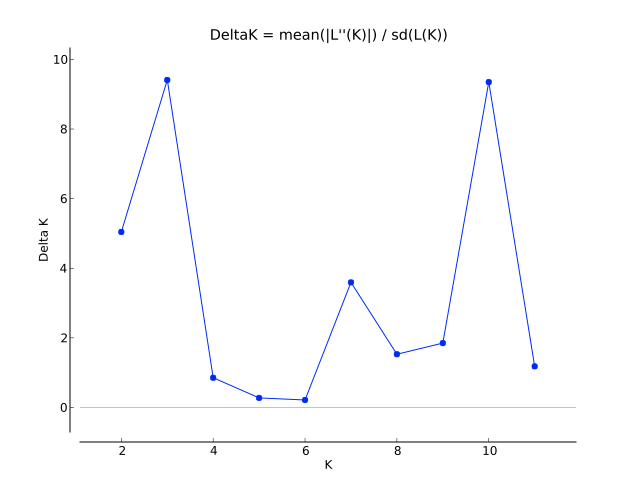


**Supplementary Material 4**

Haplotype network for 665bp of the ND4 gene.

**Supplementary Material 5**

Haplotype network for 1160bp of COI and ND4 genes concatenated.

**Supplementary Material 6**

Genetic variability of mitochondrial DNA (concatenated regions of COI and ND4).

| **mtDNA attribute** | **Population I** | **Population II** |
| --- | --- | --- |
| Number of samples (*N*) | 82 | 30 |
| Length (bp) | 1160 | 1160 |
| No. haplotypes | 20 | 13 |
| Haplotype diversity (*h*) | 0.900 (var=0.00030, sd=0.017) | 0.933 (var=0.00037, sd=0.019) |
| Nucleotide diversity (*π*) | 0.00376 | 0.00631 |
| Average no. nucleotide differences (*k*) | 4.357 (obs var=14.0759, CV=0.8638) | 7.317 (obs var=15.4245, CV=0.5412) |
| No. polymorphic sites (*S*) | 42 | 29 |
| Total no. mutations (*Eta*) | 43 | 30 |
| Tajima’s test statistic (*D*) | -1.59282 (not stat sig,0.1> P>0.05) | -0.12184 (not stat sig, P>0.1) |
| Fu’s test statistic (*Fs*) | -3.62 | -0.132 |
| Raggedness index (*r*) | 0.0138 | 0.0187 |
|  |  |  |
